# Construction of a novel electrochemical sensor based on biomass material nanocellulose and its detection of acetaminophen†

**DOI:** 10.1039/d2ra04125a

**Published:** 2022-09-28

**Authors:** Sichen Liu, Yanbo Yu, Kelu Ni, Tongda Liu, Min Gu, Yingchen Wu, Guanben Du, Xin Ran

**Affiliations:** International Joint Research Center for Biomass Materials, Yunnan Province Key Lab of Wood Adhesives and Glued Products, Southwest Forestry University Kunming 650224 China guanben@swfu.edu.cn ranxin3169@sina.com; Key Laboratory for Forest Resources Conservation and Utilization in the Southwest Mountains, Ministry of Education, Southwest Forestry University Kunming 650224 China

## Abstract

In this work, acidic sulfated cellulose nanocrystals (CNCs) were used as green carriers, and a novel composite material was synthesized and used to design sensors for paracetamol (AP) detection. There are negatively charged acidic sulfate groups on the surface of CNCs, which can enhance the electrostatic repulsion between nanoparticles, thereby increasing the stability and dispersibility of AgNPs in the system, making them less prone to agglomeration. Cationic pillar[5]arene (CP5) with a strong host–guest effect was used as a stable ligand for silver nanoparticles (AgNPs). AgNPs have good electrical conductivity and large specific surface area, which can significantly increase the peak current. In addition, CP5 has excellent supramolecular recognition performance, which can specifically recognize the guest molecule AP to form an inclusion complex, so that a large number of AP molecules are attached to the electrode surface, which is beneficial to the amplification of electrochemical signals. The prepared sensor is more attractive in terms of sensitivity and recognition performance; the host–guest binding constant was (3.37 ± 0.26) × 10^4^ M^−1^, which can be obtained with good linearity (*R*^2^ = 0.996), low detection limit (90 nM, LOD = 3*σ*/*k*, S/N = 3) and a wide linear range (0.5–500 μM). The electrochemical sensor showed good performance in quantitative analysis, stability, selectivity, reproducibility, and actual sample detection, providing high feasibility for real-time monitoring of paracetamol; it also provides a new idea for a green sensor.

## Abstract 2

In this work, acidic sulfated cellulose nanocrystals (CNCs) were used as green carriers, and a novel composite material was synthesized and used to design sensors for paracetamol (AP) detection.

## Graphical abstract

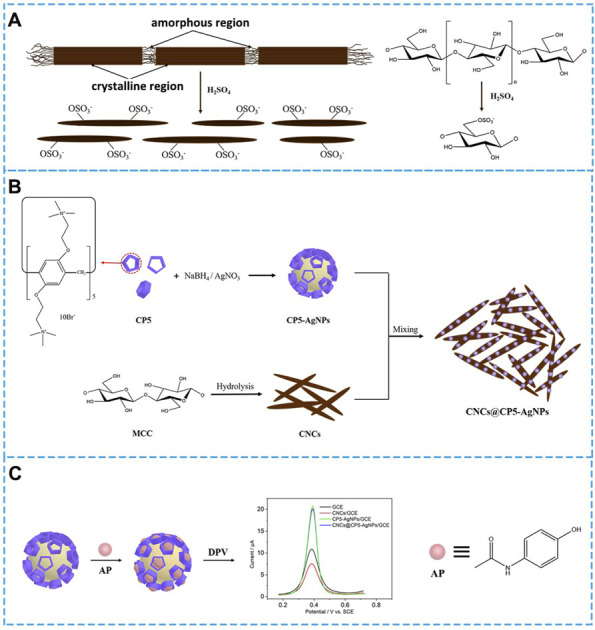

## 1. Introduction

Acetaminophen (AP) is an important analgesic that has no toxic effects on human health when taken at normal therapeutic doses. However, high doses and long-term use of AP, or concomitant use with alcohol and other drugs, may lead to rashes, liver disease, nephrotoxicity, and pancreatic inflammation.^1^ Several epidemiological studies have suggested a possible association between AP use during pregnancy and increased incidence of asthma in children. In addition, AP may re-enter the food chain through contaminated feather meal, which can be used as an additive to animal feed.^2^

The main methods currently used to detect AP are high-performance liquid chromatography,^3^ capillary electrophoresis,^4^ electrochemiluminescence,^5^ and titration analysis.^6^ Although the determination of AP using these techniques has high sensitivity and specificity, there are still some shortcomings; the assay is labor-intensive, cannot be fast and convenient, and the pre-treatment of samples is relatively complicated and cumbersome. All these factors have limited their widespread use and promotion to some extent. Based on the above considerations, the development of a simple, rapid, and accurate quantitative AP assay is imperative and of great significance, because it will directly affect clinical diagnosis, food safety, and the quality of AP based drugs. In recent years, electrochemical methods^7,8^ have been particularly widely used for the detection of AP, this method has been favored by researchers for its convenience, speed, and high sensitivity.

Electrochemical sensors are used to determine the electrical and electrochemical properties of target molecules or substances for qualitative and quantitative analysis and detection. With the functionalized modification and control of electrochemical sensors into the molecular level, electrochemical sensors with special properties are emerging and entering practical applications.^9^ The latest generation of macrocyclic subject column[n]aromatics, once reported, have been widely used,^10^ including molecular recognition, sensing detection, isomer separation and self-assembly. For many neutral small molecules and positively charged molecules, the electron cavity of column[*n*] aromatics possesses a unique supramolecular host–guest recognition effect.^11^ This makes it more strongly bound to many guest molecules and is used in large numbers as a host molecule to specifically bind the guest to the electron-deficient group, which has an important role in supramolecular chemistry due to its stability, symmetry and ease of synthesis.^12^ Recently, Jiang^13^*et al.* reported an encapsulated nanopore sensor constructed by loading the water-soluble cationic pillar[5]arene (CP5) into the nanocavity of a certain mutant (α-hemolysin) through non-covalent interactions. The detection of the highly toxic paraquat (PQ) at the single-molecule level was achieved by the superior performance of CP5 host–guest recognition. The sensor is less susceptible to environmental influences in the nanopore channel and offers greater stability and sensitivity. However, although this single structural system substantially improves the sensitivity and stability of the electrochemical sensor for recognizing small molecules, it does not reflect much in self-assembly properties, which also affects the recognition efficiency of molecules to some extent. When CP5 is attached to the surface of AgNPs,^14–17^ this effectively prevents the agglomeration of electroactive particles by self-assembly ability, which also enables the captured target molecules to have a certain spacing, thus improving the detection efficiency. And while electrochemical sensors continue to be developed, the choice of materials has also received increasing attention; cheap, portable, green, and multifunctional have become important trends in the development of sensors.

Cellulose has played an essential part in supporting human sustainable development concerns as a green renewable resource, and has been extensively explored and used in many nations. Cellulose can be hydrolyzed^18–20^ or oxidized^21,22^ to remove its amorphous part to produce cellulose nanorods of 100–300 nm in length, called cellulose nanocrystals (CNCs). The introduction of negatively charged carboxyl groups to the surface of CNCs can enhance the electrostatic repulsion between nanoparticles, thus increasing the stability and dispersion of nanomaterials in the system and making them less prone to agglomeration. Based on the effective filling structure of CNCs, a large number of primary hydroxyl groups of reactive functional groups on the surface and hemiacetal groups at the end of cyclic sugars make them superior for topological chemical modifications.^23,24^ CNCs as a nanoscale structural material has great potential for green renewable, degradable, heat resistant, and diversified modifications.^25,26^ Having received widespread attention in recent decades, CNCs have been widely used in composite materials.^27^

**Scheme 1:**
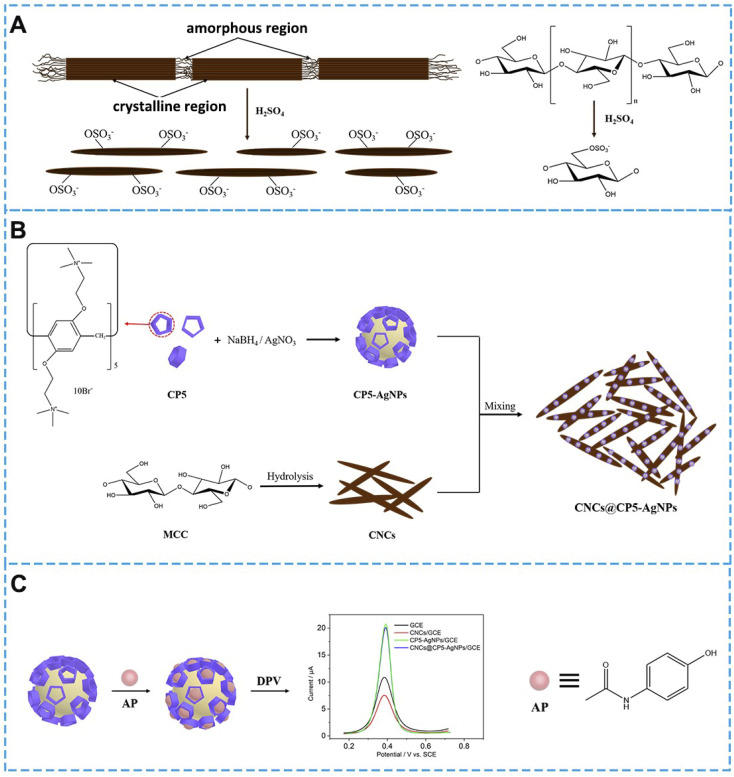
(A) Sulfuric acid hydrolysis of cellulose; (B) preparation flow chart of CP5–AgNPs, CNCs and CNCs@CP5–AgNPs; (C) electrochemical sensor of AP.

Herein, CP5 functionalized AgNPs green material cellulose nanocrystals (CNCs@CP5–AgNPs) sensors based on the integration of the latest generation of macrocyclic bodies and green material cellulose nanocrystals were successfully synthesized and used for the preparation of AP-like drug electrochemical sensors. The introduced Ag nanoparticles can maximize the capture of the target molecule by the macrocyclic body and stabilize it in its cavity, which improves the expression ability and fault tolerance of the detection process system and shows good synergy of the parts. In this experiment, CP5@Ag was prepared by a one-step reduction method, in which silver nanoparticles-cationic pillar[5]arene complexes (CP5–AgNPs) were prepared under room temperature aqueous solution conditions using the redox reaction.^28^ And the hybrid nanomaterial CNCs@CP5–AgNPs was finally fabricated by using CNCs as a green carrier and successfully complexed with CP5–AgNPs through electrostatic interaction. The excellent macrocyclic host–guest recognition and spatial steric effect of CP5 were utilized to construct the detection of paracetamol through the good electron transport properties of CP5–AgNPs and the green carrier of CNCs and its stabilizing effect on metal nanoparticles. The electrochemical sensor for the detection of paracetamol (AP) was constructed based on the excellent macrocyclic host–guest recognition and spatial steric effect of CP5 through the good electron transport properties of CP5–AgNPs and the green carrier of CNCs and its stabilizing effect on metal nanoparticles, as shown in Scheme 1.

## 2. Experimental

### 2.1 Experimental materials

Concentrated sulfuric acid (H_2_SO_4_ 98%) was purchased from Yunnan Yanglin Industrial Development Zone Shan Dian Pharmaceutical Co. Microcrystalline cellulose ((C_6_H_10_O_5_)_*n*_, *n* ≈ 220) was purchased from Shanghai Sigma Aldrich Trading Co. Silver nitrate (AgNO_3_), sodium borohydride (NaBH_4_), potassium ferricyanide trihydrate (K_4_Fe(CN)_6_·3H_2_O), potassium ferricyanide (K_3_Fe(CN)_6_), and paracetamol (C_8_H_9_NO_2_) were provided by Shanghai Adamas Reagent Co. The cationic pillar[5]arene (CP5) ((C_18_H_33_N_2_O_2_Br_2_)_5_) was prepared according to previous reported literature.^29–31^ The synthesis and characterization as shown in Fig. S1–S3.† Potassium chloride (KCl) and anhydrous ethanol (C_2_H_5_OH) were provided by Shanghai Titan Technology Co. Alumina (γ-Al_2_O_3_ 0.3 μm, 0.05 μm) was purchased from Shanghai Chenhua Instruments Co. Working water solutions were deionized water (DW), and phosphate buffer solution (PBS (pH 7.0, 0.1 M)). All chemicals were used directly without further treatment.

### 2.2 Experimental apparatus

Transmission electron microscope (TEM) images were obtained with a JEM-2100TEM instrument (Rigaku Japan). Cyclic voltammetry (CV) and differential pulse voltammetry (DPV) were performed by a three-electrode system, A CHI660E electrochemical workstation was used to investigate all electrochemical experiments (Shanghai Chenhua Instrument Co. Ltd., China). A Hitachi F-4500 fluorescence spectrophotometer was used for the fluorescence titration studies (Tokyo, Japan). The crystallinity of the samples was obtained by X-ray diffraction (XRD) (Ultima IV) (Rigaku Japan), and the surface elemental analysis was obtained on an X-ray photoelectron spectroscopy (XPS) (ESCALAB 250Xi) (Thermo Fisher Scientific, U.S.A.).

### 2.3 Preparation of CNCs, CP5–AgNPs, and CNCs@CP5–AgNPs

Referring to the method of Wu^32^*et al.*, improvements were made and CNCs were prepared. The specific steps are as follows: dilute the concentrated sulfuric acid to 64% for use, and took 0.8 g MCC and 60 mL H_2_SO_4_ (64%) in a round-bottomed flask. The temperature of the water bath was raised to 45 °C, and the round bottom flask was placed in the water bath and stirred for 60 min. Immediately after the reaction, deionized water was added to dilute it 10-fold. Used a high-speed centrifuge (speed 10 000 rpm) to centrifuge and wash 4 times, and used a rotary evaporator to evaporate the supernatant to dryness to obtain CNCs solids.

Briefly, 2 mL of deionized water, 200 μL of AgNO_3_ solution (10 mM), 1 mL of CP5 solution (0.1 mM), and 20 μL of NaBH_4_ solution (0.1 M) were added into the beaker and stirred until the color of the solution turned black gray to obtain CP5–AgNPs. An appropriate amount of solid CNCs was prepared into a suspension with a mass fraction of 0.1%. Mixed 2 mL of CP5–AgNPs and 1 mL of CNCs suspension (volume ratio 2 : 1), stirred at room temperature for 30 min to obtain CNCs@CP5–AgNPs solution, and freeze-dry to obtain a solid powder.

### 2.4 The fluorescence titration experiment

AP is insoluble in water but easily soluble in ethanol; therefore, 75.58 mg of AP solid was weighed and dissolved in 5 mL of anhydrous ethanol to prepare an AP stock solution with a concentration of 0.1 mol L^−1^. An appropriate amount of AP stock solution was diluted with 0.1 M PBS buffer (pH 7.0) to the concentration of 400 μM AP solution and set aside. Then used deionized water to prepare the CP5 solution with a concentration of 10 μM. Add 2 mL of 10 μM CP5 solution to 400 μM AP solution (10 μL each time, 10 times). After each addition, mix and stir for 5 min before recording data with a fluorescence spectrometer. The excitation wavelength (*λ*_ex_) used for the measurement was 289 nm, and fluorescence spectra were recorded in the range of 297–400 nm.

### 2.5 Preparation of modified electrode

The glassy carbon electrode (GCE) was firstly polished with 0.3 and 0.05 μm Al_2_O_3_ powders then rinsed with tap water, anhydrous ethanol, and deionized water respectively after about 180 times on a chamois leather and left to dry at room temperature. 6 μL of CNCs@CP5–AgNPs solution was pipetted onto the air-dried GCE. For comparison, 0.1% CNCs and CP5–AgNPs-modified electrodes were prepared using the same method. Allow drying at room temperature then perform electrochemical tests.

### 2.6 Electrochemical measurements

Three-electrode system consisting of a glassy carbon electrode (working electrode), a platinum electrode (auxiliary electrode), and a saturated glycerol electrode (reference electrode) was used for electrochemical testing. The AP stock solution was diluted with pH 7.0 PBS buffer (0.1 M) to various concentrations (as electrolyte solution) for subsequent cyclic voltammetry (CV) and differential pulse voltammetry (DPV) tests. CV sweep range: −0.2–0.8 V, sweep rate 100 mV s^−1^ (for electrolyte solution of 2 mM [Fe(CN)_6_]^3−/4−^, sweep range: −0.2–0.6 V; sweep rate 50 mV s^−1^). DPV sweep range: 0.2–0.8 V, pulse width 0.05 s, sampling width 0.02 s. The electrochemical impedance spectra (EIS) were tested for all frequencies in the range 10^−2^–10^5^ Hz with an amplitude of 0.005 V. The electrolyte solution was 2 mM [Fe(CN)_6_]^3−/4−^ prepared with 0.1 M KCl. All tests were performed at room temperature and all electrode potentials were relative to SCE.

The actual performance of the sensor was evaluated using commercially available acetaminophen tablets. One AP tablet (300 mg) was ground into a powder, since AP is easily soluble in anhydrous ethanol, hardly soluble in water. Therefore, it was first dissolved with a certain amount of anhydrous ethanol and then diluted with PBS (pH 7.0, 0.1 M) to the desired concentration for electrochemical quantification. Finally, the relative standard deviation (RSD) and recovery were calculated.

## 3. Results and discussions

### 3.1 Characterization of CNCs, CP5–AgNPs, and CNCs@CP5–AgNPs

**Fig. 1:**
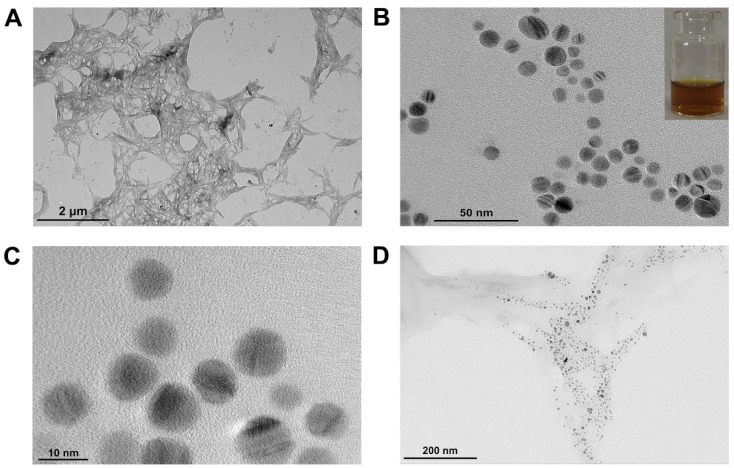
TEM diagram of (A) CNCs; (B) and (C) CP5–AgNPs (illustration: CP5–AgNPs is yellow in the sunlight); (D) CNCs@CP5–AgNPs.

It can be seen that the CNCs prepared by sulfuric acid hydrolysis are rod-shaped (Fig. 1A), with a length: of 100–400 nm; diameter: of 3–12 nm, which are consistent with those reported in the literature^33^ and in accordance with the size characteristics of CNCs. Fig. S4A† shows the FT-IR spectra of CNCs and MCC, it can be seen that CNCs showed strong absorption peaks at 3352 cm^−1^, 2913 cm^−1^; MCC at 3349 cm^−1^, 2899 cm^−1^, which are the peaks of stretching vibrations from O–H and C–H groups, respectively. The stretching vibration bands of O–H in CNCs are narrower than those of MCC, and the positions of the peaks are shifted to the left, indicating that the hydrogen bonds within the CNCs molecules become stronger. The absorption peak at 1640 cm^−1^ is a stretching vibration of the water-absorbing O–H group caused by the abundant hydrophilic groups on the surface of cellulose, which indicates that CNCs have a strong ability to adsorb water molecules. Both have similar characteristic absorption peaks at 1430 cm^−1^, 1163 cm^−1^, 1114 cm^−1^, and 898 cm^−1^ attributed to C–H in the methyl group, C–C backbone, stretching vibrations of the intramolecular C–O, and wobbling vibrations of β-1,4-glycosidic bond, respectively, corresponding to the characteristic absorption peaks of cellulose *I*_β_. It can be seen that the acid hydrolysis process did not destroy the basic chemical structure of cellulose, CNCs still had characteristic peaks similar to those of cellulose. CNCs showed a new absorption peak at 1760 cm^−1^, corresponding to stretching vibration of C=O. Hydrolysis process selectively oxidizes the hydroxyl group at the C6 position of cellulose to the carboxyl group.

Fig. S4B† shows the XRD curves of CNCs and MCC, the positions of the diffraction peaks of both remain the same, the four diffraction peaks located at 15.18°, 16.41°, 22.42°, and 34.58° correspond to (*110), (110), (200), and (004) crystallographic planes of cellulose type I, respectively.^34^ The trough region of 2*θ* = 18°, the amorphous region of cellulose, has higher activity compared to the crystalline region. Therefore, acid can penetrate and remove the amorphous zone better without affecting the acid-insoluble crystalline zone. Highly crystalline zones are more resistant to acid hydrolysis than amorphous zones,^35^ they are easily separated from the hydrolysate. The main activity of the acid is the release of hydrogen ions (H_3_O^+^), and can simultaneously sever the glycosidic bonds within the cellulose nanofiber bundles and amorphous chain segments, eventually leaving cellulose nanocrystals. This proves that acid hydrolysis occurs in the amorphous region of MCC and does not destroy its crystal structure, which preserves its properties. This is consistent with the results of IR spectroscopy tests, so in this paper, CNCs were successfully prepared using sulfuric acid hydrolysis of MCC.

The AgNPs were prepared by reducing AgNO_3_ with NaBH_4_, functionalized composite nanomaterials CP5–AgNPs were made by adding CP5 as a stabilizer. Microscopic morphology, distribution state, and particle size of CP5–AgNPs were observed by transmission electron microscopy. As shown in Fig. 1B and C, CP5–AgNPs were spherical with uniform particle distribution, obvious lattice, and good dispersion. The size was about 10.56 ± 1.62 nm, and the solution was yellow under daylight. However, the AgNPs prepared without CP5 are seriously aggregated (Fig. S5†), which indicates that CP5 has the advantage to prevent the aggregation of AgNPs. The composite material CNCs@CP5–AgNPs was made by mixing CP5–AgNPs with CNCs solution with a mass fraction of 0.1% in the ratio of 2 : 1 by volume, from the TEM images Fig. 1D, it can be seen that the silver nanoparticles were dispersed on the nanocellulose crystals in an orderly and uniform manner by electrostatic interaction, and there was no obvious agglomeration. In addition, CNCs being a natural and green carrier, the carboxyl groups introduced on their surface due to hydrolysis can also adsorb metal particles, which can prevent the agglomeration of CP5–AgNPs to a certain extent, play the role of co-stabilizing silver nanoparticles with CP5.

The successful composite of CP5–AgNPs and CNCs and the successful preparation of silver nanoparticles can be seen from the characterization results of XRD and XPS. The eight distinct diffraction peaks in Fig. S4C:† 14.92°, 16.52°, 22.71°, 34.58°, 38.12°, 44.34°, 64.50°, 77.30° correspond to the cellulose type I of CNCs and (111), (200), (220), (311) crystallographic planes (JCPDS file no. 04-0783)^36^ of CP5–AgNPs, respectively. The combination of the two did not change or generate any chemical groups that affected the generation of silver nanoparticles.

**Fig. 2:**
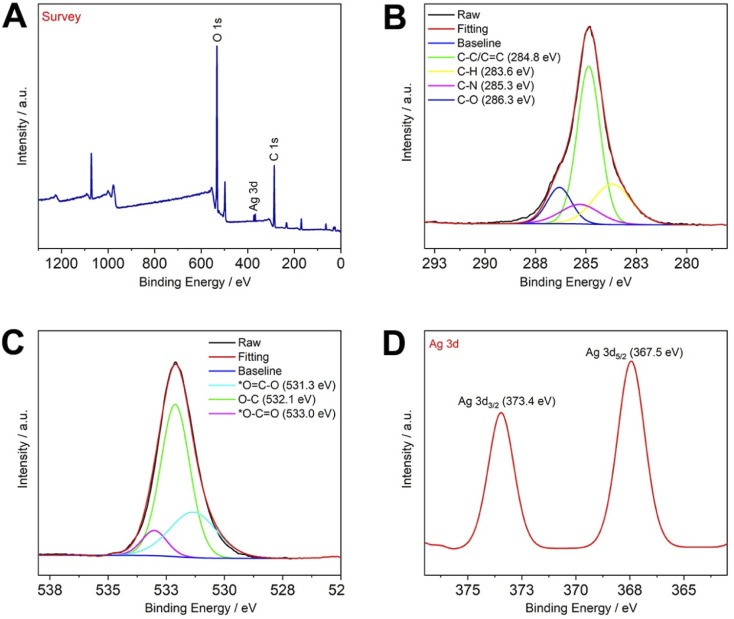
(A) XPS spectrum of CNCs@CP5–AgNPs; high-resolution XPS of (B) C 1s; (C) O 1s; (D) Ag 3d.

The same can be demonstrated by XPS, as shown in Fig. 2, where the XPS spectra indicate the presence of C, O, and Ag as major elements in CNCs@CP5–AgNPs. Among them, the high-resolution XPS spectrum of C 1s shows that carbon atoms are mainly bound in four forms, C–C/C=C (284.8 eV), C–H (283.6 eV), C–N (285.3 eV), C–O (286.3 eV), while O 1s shows three binding types, namely *O=C–O (531.3 eV), O–C (532.1 eV), *O–C=O (533.0 eV). The high-resolution XPS spectrum of Ag 3d contains two peaks, one at 367.5 eV (Ag 3d_5/2_) and the other at 373.4 eV (Ag 3d_3/2_), which is consistent with that reported in the relevant literature.^37^ The above conclusion can also be illustrated by comparing the XPS spectra of the control group (Fig. S6 and S7†). Therefore, in this paper, silver nanoparticles were successfully synthesized and successfully compounded with CNCs, thus dispersibility of CP5–AgNPs can be increased and used to develop functional nanomaterials based on CNCs with excellent properties.

### 3.2 Host–guest recognition studies

**Fig. 3:**
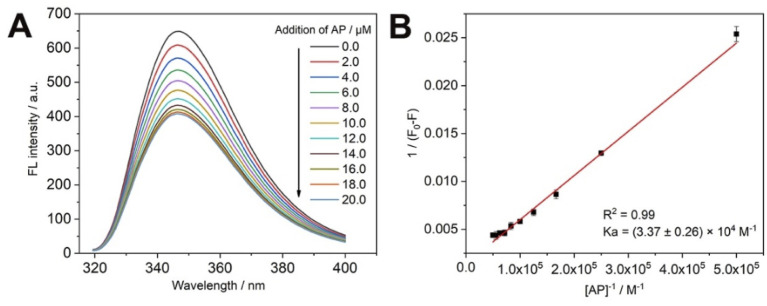
(A) Fluorescence quenching of CP5 by different concentrations of AP; (B) the double reciprocal curve between the fluorescence intensity variable of CP5 and the concentration of AP.

As shown in Fig. 3A, the fluorescence intensity of the main body (CP5) gradually decreases when the guest (AP) is added to the main body (CP5) in successive drops, thus the recognition ability of CP5 to AP is investigated. In this paper, we used the fact that CP5 has the maximum fluorescence intensity at the excitation wavelength of 289 nm to find the binding constant with AP. Where CP5 is a constant quantity, AP is added to CP5 as a variable in successive drops to obtain the corresponding fluorescence spectra. The linear relationship between the concentration of AP solution and fluorescence intensity of CP5 was made using the double inverse method (Fig. 3B). The magnitude of the principal-object binding constant *K*_a_ can be found using the equation: 1/(*F*_0_ − *F*) = 1/[(*F*_∞_ − *F*_0_) × *K*_a_ × *C*_AP_] + 1/(*F*_∞_ − *F*_0_), where *F* is the fluorescence intensity of CP5 at different AP concentrations, *C*_AP_ is the concentration of AP, 1/[(*F*_∞_ − *F*_0_) × *K*_a_] is the slope of the double inverse curve, 1/(*F*_∞_ − *F*_0_) is an intercept of the double inverse curve. The resulting CP5/AP binding constant is (3.37 ± 0.26) × 10^4^ M^−1^. Therefore, CP5 can specifically recognize AP molecules for quantitative detection of AP.

### 3.3 Characterization of different modified electrodes

Comparing the CV curves of the four electrodes GCE, CNCs/GCE, CP5–AgNPs/GCE, CNCs@CP5–AgNPs/GCE in 2 mM [Fe(CN)_6_]^3−/4−^ solution (Fig. S8A†), it can be found that peak current of the electrode loaded with CNCs in this solution is smaller than that of the bare electrode, which is since CNCs are highly hydrophilic. The addition of CP5–AgNPs has a significant effect on the increase of oxidation and reduction of peak currents, indicating that AgNPs have excellent electrical conductivity and play a key role in increasing the specific surface area of the electrode. Although the peak current was reduced compared to that of CP5–AgNPs/GCE, the reduction is not large. Therefore, prepared CNCs@CP5–AgNPs/GCE can accelerate electron transport, and the electrode has excellent electrochemical performance and can be used for further detection experiments.

In addition, the interfacial properties of the electrode can be studied by testing the AC impedance spectra of the electrode in the electrolyte solution, changing the impedance value reflects the different modification processes on the electrode surface. Resistance to electron transfer in the modified layer is obtained by the hindrance of electron transfer by the redox probe molecule [Fe(CN)_6_]^3−/4−^. The diameter of the arc is proportional to the magnitude of charge transfer resistance, a decrease in diameter indicates a decrease in resistance. Electrode impedance values of modified CP5–AgNPs are significantly reduced compared to GCE (Fig. S8B†), which are approximately a straight line in a frequency range. This is due to the excellent electrical conductivity of silver nanoparticles, which accelerates electron transfer rate, increases conductivity, and enhances electrical conductivity. However, composite CNCs@CP5–AgNPs modified electrode shows another slight increase in impedance value of about 180 Ω, which is consistent with the results of CV curves described above. Poor conductivity of CNCs hinders electron transfer, which indirectly proves the successful composite of CNCs with CP5–AgNPs, two forms of the composite nanomaterial CNCs@CP5–AgNPs.

Active surface area of the different modified electrodes can be found according to the Randles–Sevcik equation: *I*_pa_ = 2.69 × 10^5^*n*^3/2^*AcD*^1/2^*v*^1/2^. Where *I*_pa_ is peak current, *n* is several electron transfers, *A* is the surface area of the electrode, *c* is [Fe(CN)_6_]^3−/4−^ concentration, *D* is diffusion coefficient, *v* is sweep speed. Effective surface areas of GCE and CNCs@CP5–AgNPs/GCE are calculated to be 0.106, and 0.163 cm^2^ respectively. This indicates that after modification of the hybrid material, effective area of the electrode is increased, which increases the enrichment capacity of the electrode surface for AP, and facilitates the transfer of electrons from electroactive substances.

### 3.4 Optimization of measurement conditions

**Fig. 4:**
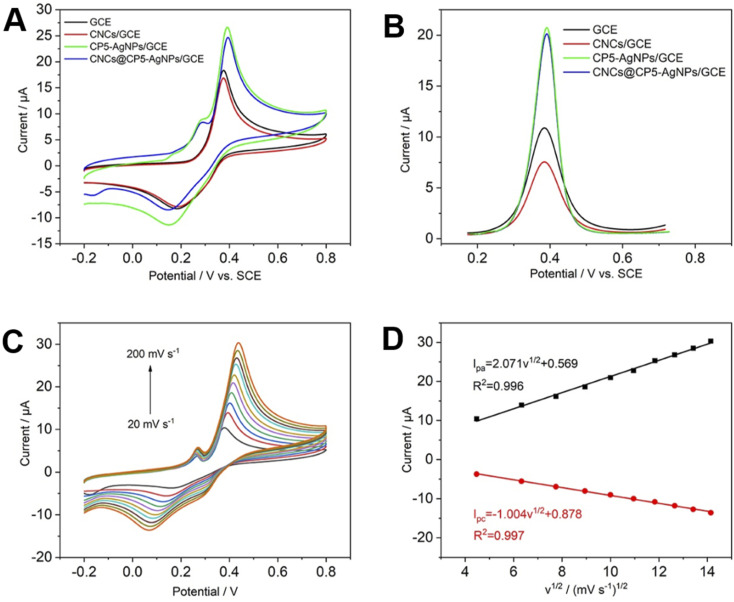
(A and B) CV curves and DPV curves of different electrodes in 500 μM AP solution, respectively; (C) CV curves of CNCs@CP5–AgNPs/GCE in 500 μM AP solution at different scanning rates; (D) current response *vs.* square root of scan rate.

As shown in Fig. 4A and B, the electrochemical behavior of AP was investigated by CV and DPV curves, from which the possibility of building this electrochemical platform was determined. A 500 μM AP solution was prepared with 0.1 M PBS (pH 7.0), CV and DPV tests were performed with GCE, CNCs/GCE, CP5–AgNPs/GCE, CNCs@CP5–AgNPs/GCE, respectively. From the figure, the peak currents of the electrodes modified with CP5–AgNPs and CNCs@CP5–AgNPs are significantly larger than those of the bare electrodes. Although the addition of CNCs will have some influence on the electrochemical properties of electrodes, the comparison shows that this small difference will not have a large impact on the whole experiments conducted. It also shows that silver nanoparticles with good electrical conductivity and a large specific surface area can increase the peak current significantly, coupled with the excellent supramolecular recognition property of CP5, which can specifically recognize the guest molecule AP and thus form an inclusion compound, so that a large number of AP molecules can be attached to the electrode surface. Conducive to the amplification of electrochemical signals. In the CV curves, CNCs@CP5–AgNPs/GCE can be observed as a pair of obvious redox peaks with the outgoing positions *E*_pa_ 391 mV and *E*_pc_ 150 mV, prepared modified electrode is favorable for the reversible electrochemical reaction of AP, as shown in Fig. S9.†

The significant increase in peak current and clear redox peaks indicate that CNCs@CP5–AgNPs modified electrode not only has high electrochemically active surface area but also has good electrocatalytic activity, which is an ideal property for the development of electrochemical sensors. The effect of different scan rates on the electrochemical behavior of AP was investigated. The CNCs@CP5–AgNPs modified electrodes were placed in an electrolyte solution (500 μM, pH 7.0) with cyclic voltammetry, different sweep rates were set and CV curves were recorded. As seen in Fig. 4C, in the range of 20–200 mV s^−1^, with scan rate increases, oxidation peak current (*I*_pa_) and reduction peak current (*I*_pc_) also increase continuously and are accompanied by a rightward shift of the peak potential. A plot with the peak current magnitude as the vertical coordinate and the square root of the scan rate as the horizontal coordinate shows a linear between the two relationships. The linear regression equation is *I*_pa_ (μA) = 2.071*v*^1/2^ (mV s^−1^)^1/2^ + 0.569 (*R*^2^ = 0.996) and *I*_pc_ = −1.004*v*^1/2^ (mV s^−1^)^1/2^ + 0.878 (*R*^2^ = 0.997) Fig. 4D. It indicates that the electrochemical reaction of AP at the modified electrode is a classical diffusion-controlled process.^38,39^ At slower sweep speeds, the oxidation peak current of AP is small and sensitivity is low; as the sweep speed increases, peak current increases and peak potential difference increases, reversibility of the electrode becomes poor. Therefore, 100 mV s^−1^ is the best sweep speed in this system.

The pH and enrichment conditions of the electrolyte solution can have a large impact on the electrochemical behavior of AP at modified electrodes. To ensure the high sensitivity of this sensor in practical detection, these two key factors were investigated using DPV to obtain the optimal experimental conditions. As shown in Fig. S10A,† PBS with pH 5.0–9.0 was prepared using different ratios of disodium hydrogen phosphate and sodium dihydrogen phosphate, the value of the oxidation peak current of AP increased when the pH increased from 5.0 to 7.0, while the oxidation peak current gradually decreased when the pH increased from 7.0 to 9.0. It can be concluded that the optimum pH of the electrolyte solution is 7.0.

AP oxidation peak potential also shifts negatively with increasing pH, linear regression equation is: *E*_pa_ (V) = 0.851 − 0.063pH (*R*^2^ = 0.996). It shows the involvement of protons in the reaction process, from the equation *E*_p_ = *K* − (*mRT*/*nF*)ln[H^+^] (*m* is the number of protons and *n* is the number of electrons), we can get *E*_p_ = *K* − (0.057*m*/*n*)pH, calculation by substituting the relevant data gives *m* = 1.97 ≈ 2. Therefore, the reaction of AP at the electrode is two electrons and two protons are involved in a quasi-reversible process. It can be seen that the peak current of the oxidation peak increases and then decreases in the range of enrichment potential 0.0–0.7 V, with maximum current at 0.3 V (Fig. S10B†). In summary, optimal pH and enrichment potential were set to 7.0 and 0.3 V, respectively, as a way to improve the electrical signal and sensitivity of detecting the guest AP.

### 3.5 Quantitative detection analysis of AP

**Fig. 5:**
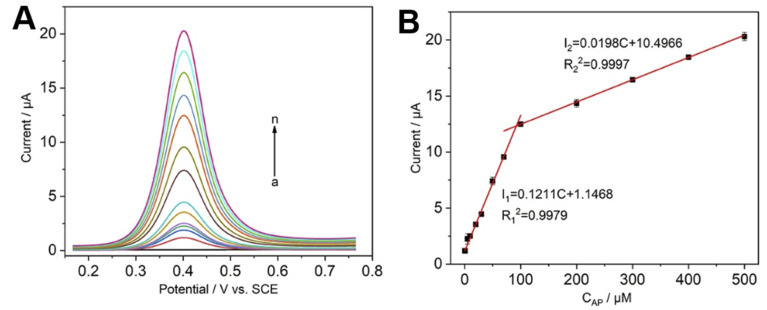
(A) DPV response curve of CNCs@CP5–AgNPs/GCE to different concentrations of AP solution (*a*–*n*: 0, 0.5, 1, 5, 10, 20, 30, 50, 70, 100, 200, 300, 400, 500 μM); (B) AP concentration correction curve.

After the optimal experimental conditions were determined, the quantitative detection experiments of the guest AP were carried out using DPV, results are shown in Fig. 5A. The oxidation peak current increased with the increase of solution concentration when the electrode modified with CNCs@CP5–AgNPs was placed into different concentrations of AP solution, and the solution concentration of AP was linearly proportional to the oxidation peak current (Fig. 5B). In the concentration ranges of 0.5–100 μM and 100–500 μM, respectively, linear regression equations were *I*_1_ (μA) = 0.1211*C* (μM) + 1.1468; *I*_2_ (μA) = 0.0198*C* (μM) + 10.4966, with correlation coefficients of 0.9979, 0.9997, the detection limit was 90 nM (LOD = 3*σ*/*k*, where *σ* is RSD of the blank sample, *k* is the slope of the curve, S/N = 3) based on the linear equation (*I*_1_ (μA) = 0.1211*C* (μM) + 1.1468, *R*^2^ = 0.9979) in the low concentration range (0.5–100 μM). Compared to other electrode materials (Table 1), such as carbon nanotubes (CNTs), nanoparticles (NPs), graphene, biomass materials, and supermolecule, the CNCs@CP5–AgNPs modified electrode for the detection of PA showed a low detection limit and wide linear range, and the detection environment (pH 7.0) was close to that of human blood. More importantly, cellulose as a carrier is green and environmentally, which is beneficial to improve the stability of the materials, reduce the risk of nanoparticles leaching from the surface of the sensor, improve the recovery rate of nanoparticles, and have practical value.

**Table 1:** Comparison of electrochemical methods for quantitative detection of AP

Electrode	pH	Method	Linear range (μM)	LOD (nM)	Reference
SWCNT/GCE	7.0	CV	0.2–150	120	40
f-MWCNT/GCE	8.0	DPV	3–200	600	41
np-MnFe_2_O_4_/GP	6.0	CV/ASV	3–160	300	42
rGO/GCE	7.2	CV	0.05–45	465	43
DLC/VAMWCNT	2.0	SWV	0.997–36.7	334	44
Poly/AHNSA/GCE	4.5	CV	10–125	450	45
P–NC/GCE	7.0	CV	3–110	500	46
AgNP-xGnP/GCE	7.5	CV/DPV	4.98–33.8	85	47
CS/Ag–Pd–rGO/GCE	8.0	CV/DPV	0.5–300	230	48
β-CD/GCE	5.5	CV	0.1–80	97	49
CNCs@CP5–AgNPs/GCE	7.0	DPV	0.5–500	90	This work

### 3.6 Selectivity and reproducibility studies

Selectivity and reproducibility are important indicators to evaluate the performance of electrochemical sensors. In a similar physiological environment, DA and AA, which have similar oxidation peak potentials to AP, as well as hydroquinone and nitrobenzene phenol, which have similar structures to AP, was specifically selected for this experiment, and the presence of interfering substances would easily lead to the overlapping of oxidation peaks thus making it difficult to read the peak magnitude of the target substances. It can be seen from Fig. S11,† 10-fold AA, DA, UA, BSA; 20-fold glucose, sucrose, cysteine, alanine, tryptophan, hydroquinone, *p*-nitrophenol, 100-fold NaCl, and KOH did not have much effect on the quantitative detection of AP, and the peak currents obtained were almost the same as those of the samples containing only AP. This is due to the stronger binding ability of AP to CP5 than to the interferents, indicating the good selectivity of the sensor and its resistance to interference. Further, the reproducibility of the sensor was investigated, using 12 electrodes modified with CNCs@CP5–AgNPs for the test and comparing the electrical signal of AP, yielding a relative standard deviation (RSD) of 3.5%, which is also a significant advantage of this experimental design in terms of reproducibility.

### 3.7 Analysis of AP in human urine

To investigate the feasibility of the sensor in practical applications, the actual performance of the sensor was evaluated in this experiment using paracetamol drug samples. Firstly, one paracetamol tablet (300 mg) was ground into powder, dissolved in a certain amount of anhydrous ethanol, and then transferred into a volumetric flask and diluted with 0.1 M PBS (pH 7.0) in fixed volume to the working concentration range for electrochemical determination. The results showed that the mean assayed concentration of paracetamol was 289 mg per tablet, which was consistent with the tablet content provided by the manufacturer with minimal error. Further, to verify the applicability of the CNCs@CP5–AgNPs/GCE method, the content of paracetamol in human urine samples was determined using a standard spiking method. Before analysis, urine samples were diluted 50-fold with PBS pH 7.0 to reduce the complexity of the matrix. The results of the assay after adding a certain concentration of AP to a standard urine sample are presented in Table 2. A relative standard deviation between 1.78–2.03% and a recovery of 99.02–100.51%. The sensor has the potential to detect real samples with stable results and small errors in multiple assays under complex conditions.

**Table 2:** Experiment on recovery of AP in human urine samples by adding standard (*n* = 6)

Sample	Added (μM)	Found (μM)	RSD (%)	Recovery (%)
1	20	20.102	1.78	100.51
2	50	49.906	1.98	99.81
3	100	99.021	2.03	99.02

## 4. Conclusions

In this work, CNCs were prepared by sulfuric acid hydrolysis and used as green carriers to compound with CP5-functionalized silver nanoparticles to prepare an electrochemical sensor (CNCs@CP5–AgNPs) capable of specific recognition and quantitative detection of AP. And this system can maximize the capture of target molecules and stabilize them in their cavities, which improves the expression ability and fault tolerance of the detection process system and shows a good synergy of all parts. It not only enhances the effective recognition of target molecules, but also improves the loading rate, electrical signal transmission and dispersion performance in the environment. Comparing with the same type of assay, it was found that the assay has the advantages of low detection limit, wide linear range, high sensitivity and high anti-interference ability. Reproducibility, stability and experimental results of actual sample testing further demonstrate that the sensor has potential applications.

## Conflicts of interest

There are no conflicts to declare.

## Supplementary Material

RA-012-D2RA04125A-s001
