# Supplementary material for: Construction of a novel electrochemical sensor based on biomass material nanocellulose and its detection of acetaminophen
Source: RSC Adv. 2022 Sep 28;12(43):27736–45. doi: 10.1039/d2ra04125a (PMC9516959; doi:10.1039/d2ra04125a)
Supplement: RA-012-D2RA04125A-s001 [file RA-012-D2RA04125A-s001.pdf]

Electronic Supplementary Material (ESI) for RSC Advances.
This journal is © The Royal Society of Chemistry 2022

Supporting Information

Construction of a novel electrochemical sensor based on biomass material nanocellulose and its detection of acetaminophen

Sichen Liu[a], Yanbo Yu[a], Kelu Ni[a], Tongda Liu[a], Min Gu[a], Yingchen Wu[a], Guanben Du[a,b,*], Xin Ran[a,b,*]

[a] International Joint Research Center for Biomass Materials, Yunnan Province Key Lab of Wood Adhesives and Glued Products, Southwest Forestry University, Kunming 650224, China

[b] Key Laboratory for Forest Resources Conservation and Utilization in the Southwest Mountains, Ministry of Education, Southwest Forestry University, Kunming 650224, China

***Corresponding Authors**

*E-mail: guanben@swfu.edu.cn (G. Du); ranxin3169@sina.com (X. Ran)

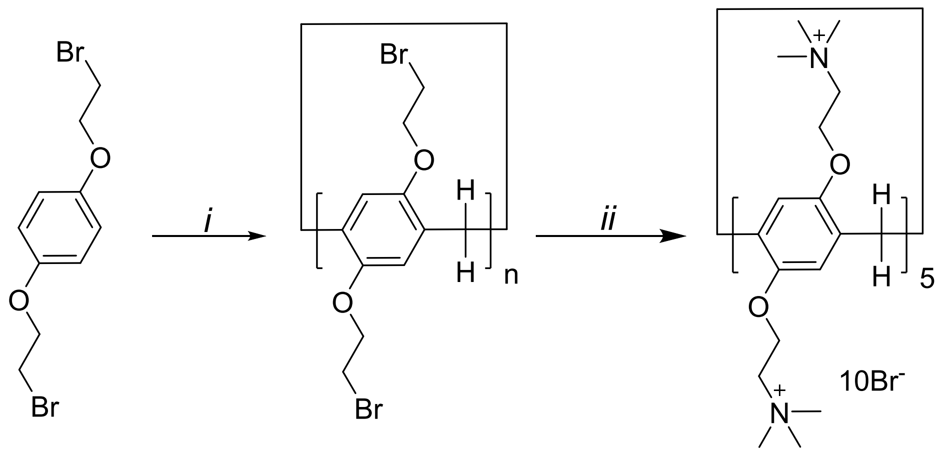

1 **2** **CP5**

Fig.S1 The synthetic route of CP5.

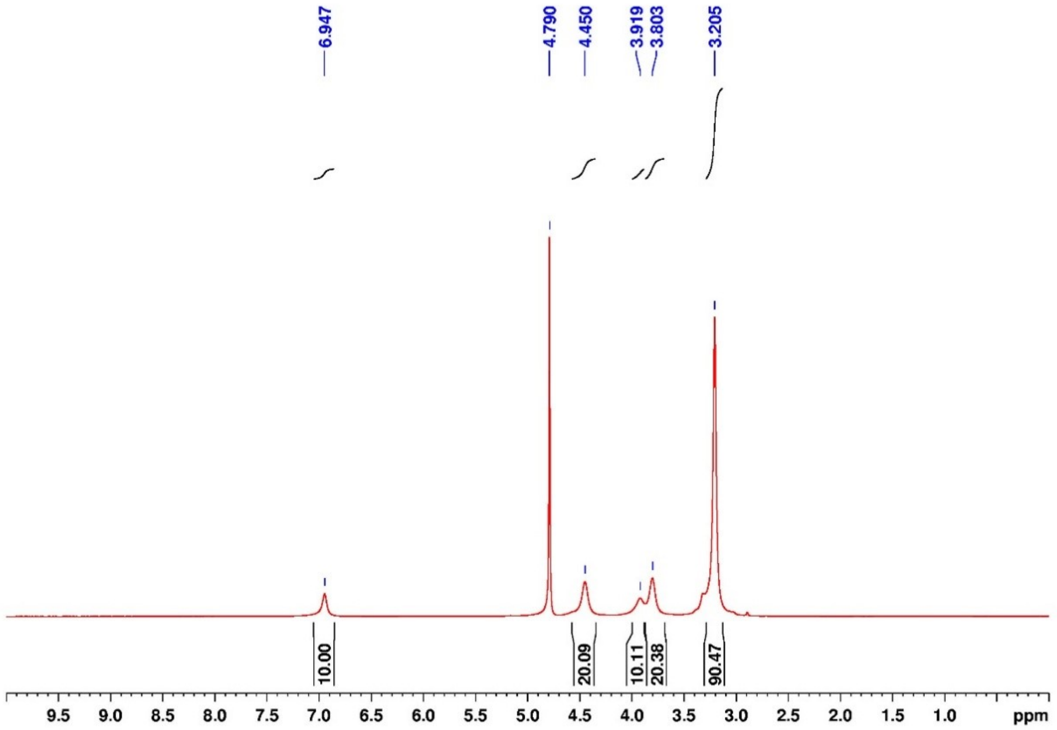

Fig.S2 ^{1}H NMR spectrum (400 MHz, D_2O, rt) of CP5.

^{1}H NMR (400 MHz, D_2O, rt) δ (ppm): 6.947 (s, 10H, 10Ar**H**), 4.450 (s, 20H, 10ArOC**H**$_2$), 3.919 (s, 10H, 5ArC**H**$_2$Ar), 3.803 (s, 20H, 10ArOC**H**$_2$), 3.205 (s, 90H, 30NC**H**$_3$).

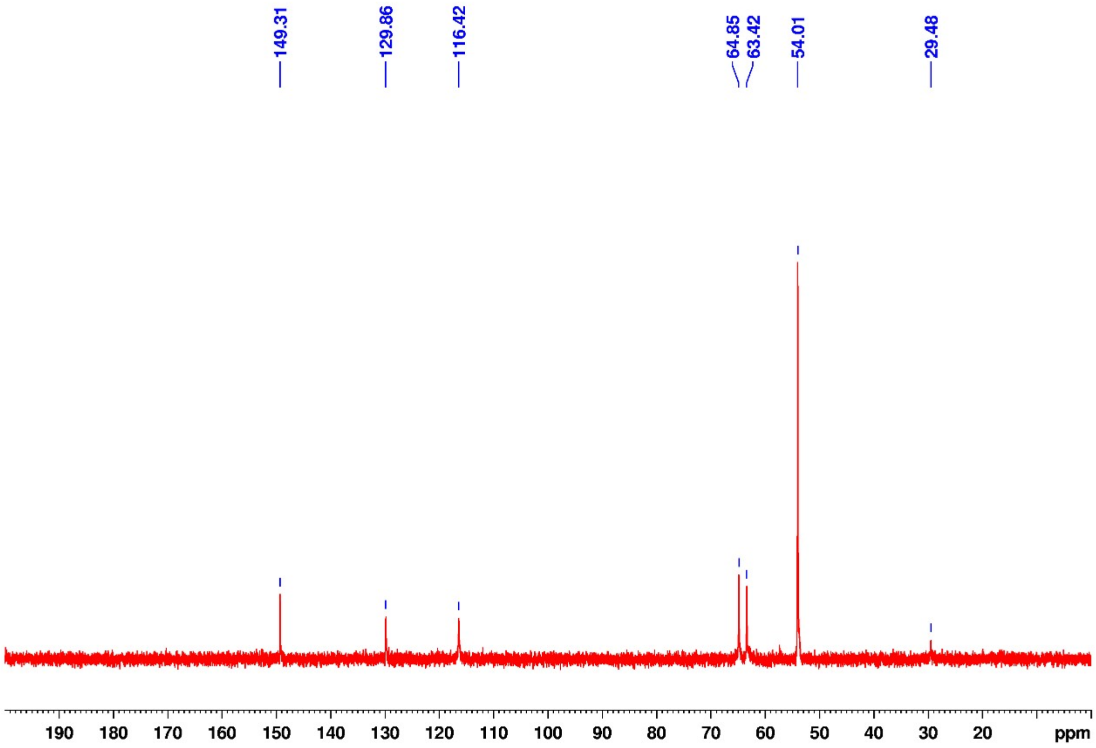

Fig.S3 ^{13}C NMR spectrum (100 MHz, D_2O, rt) of CP5.

^{13}C NMR (100 MHz, D_2O, rt) δ (ppm): 149.31, 129.86, 116.42, 64.85, 63.42, 54.01.

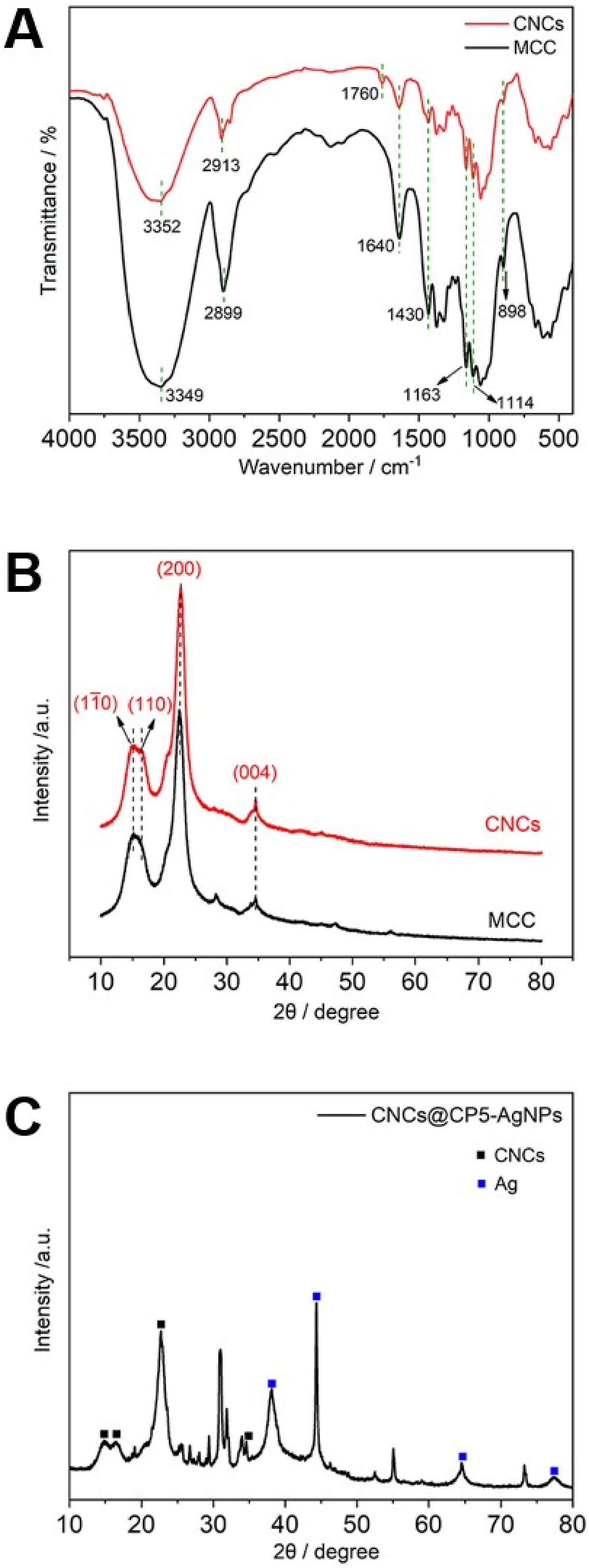

Fig.S4 (A) FT-IR of CNCs and MCC; XRD spectrum of (B) CNCs, MCC; (C) CNCs@CP5-AgNPs.

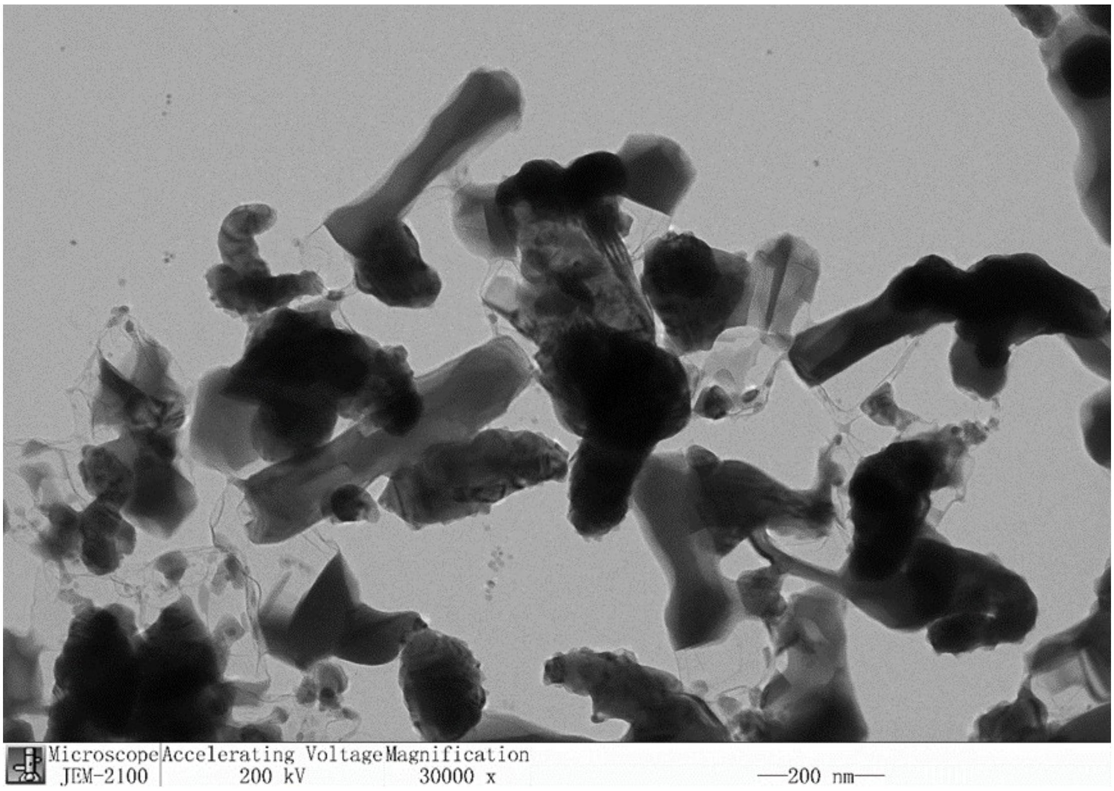

Fig S5 The TEM image of AgNPs was prepared in the absence of CP5.

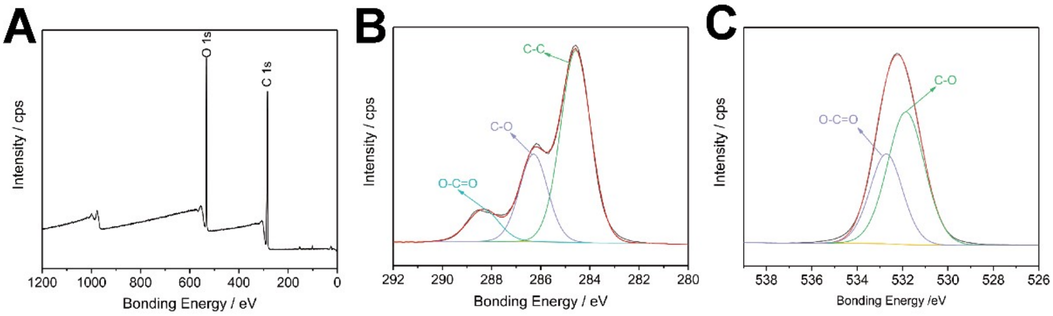

Fig S6 (A) XPS spectrum of CNCs; high-resolution XPS of (B) C 1s; (C) O 1s.

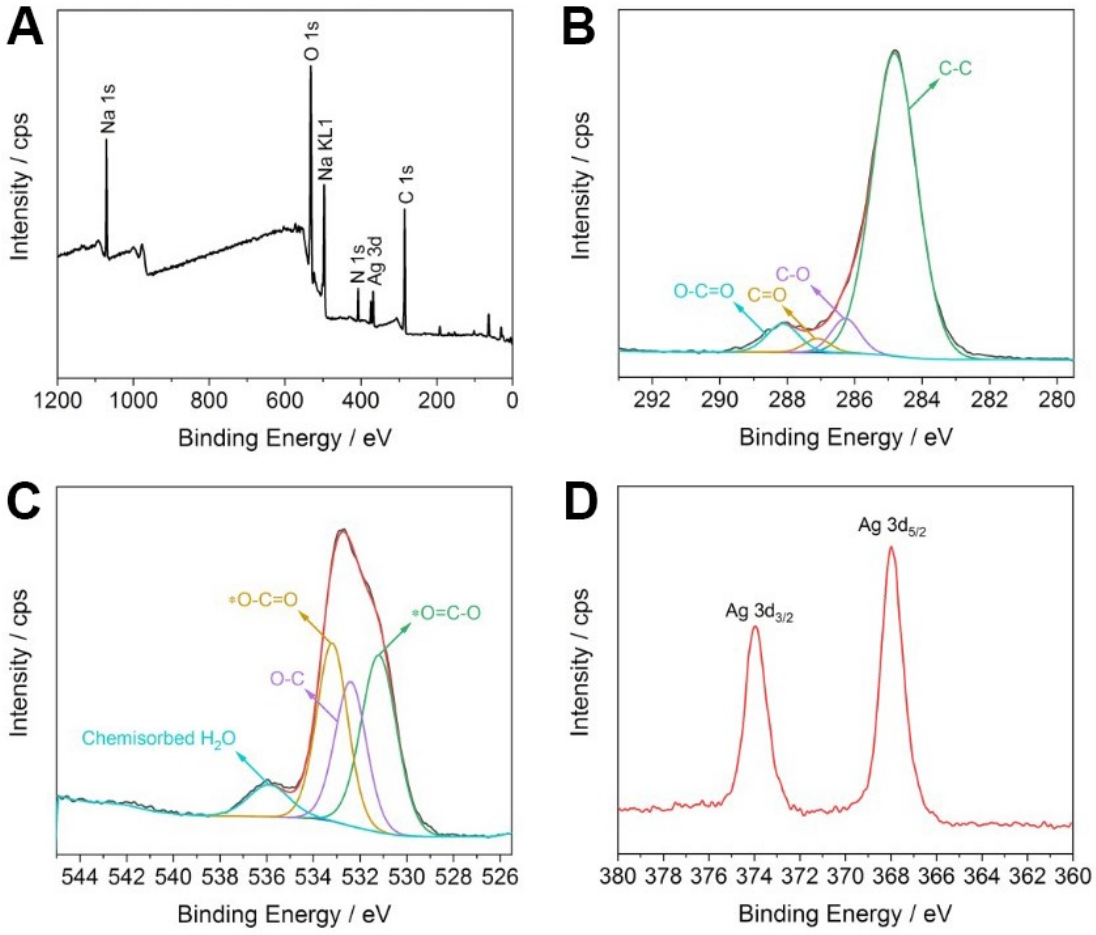

Fig S7 (A) XPS spectrum of CP5-AgNPs; high-resolution XPS of (B) C 1s; (C) O 1s; (D) Ag 3d.

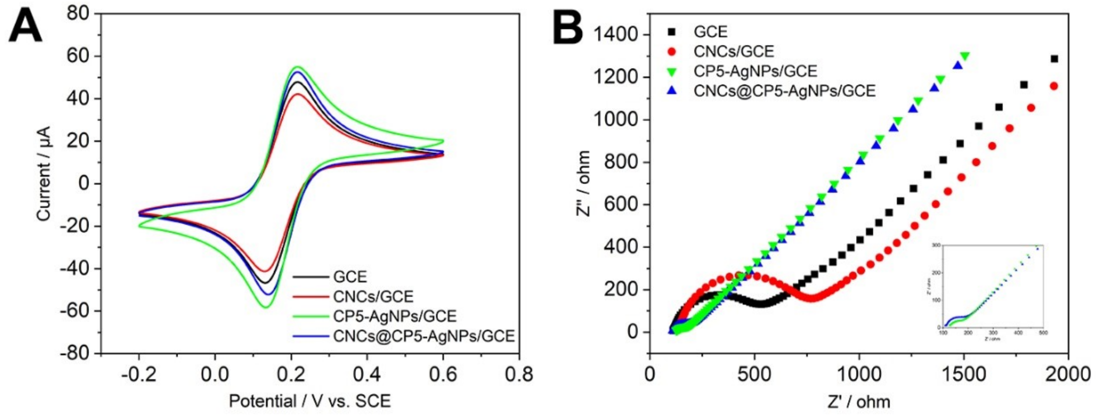

Fig.**S8** CV curves of different electrodes in 2 mM $[Fe(CN)_6]^{3-/4-}$ solution(A), EIS spectrum(B).

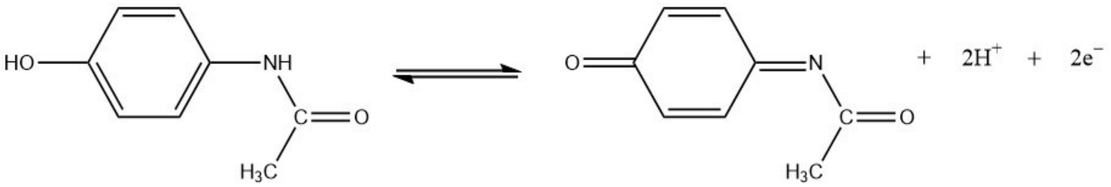

Fig.**S9** Reaction mechanism of AP on electrode.

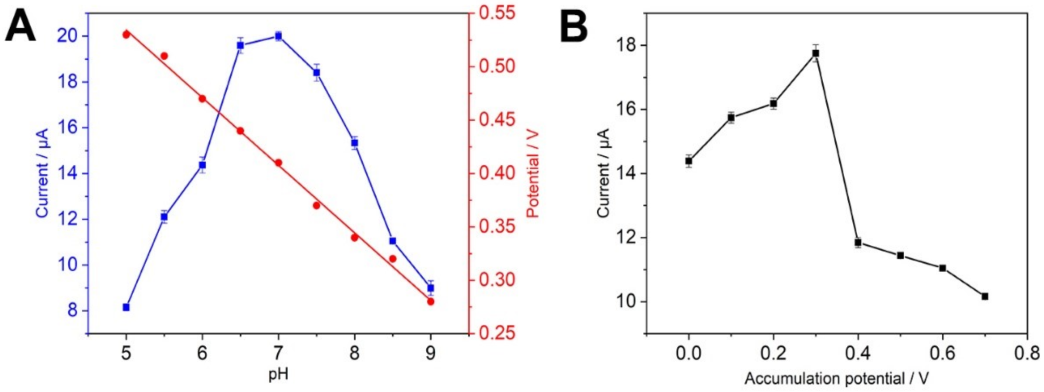

Fig.**S10** Effect of pH on peak current and potential of AP(A), effect of accumulation potential on peak current of AP(B).

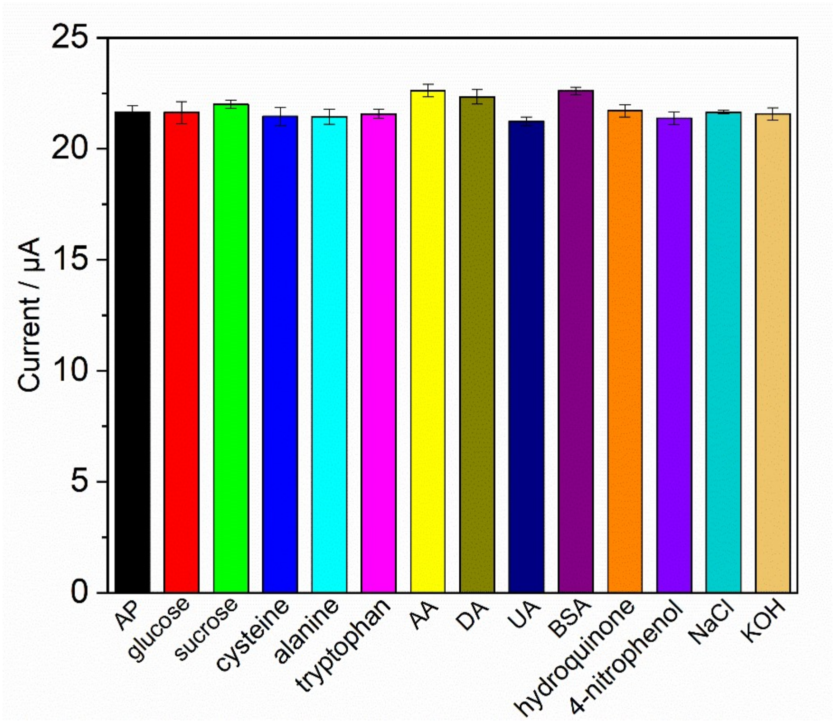

Fig.**S11** Selective experiment of AP.
